# GIGANTUS1 (GTS1), a member of Transducin/WD40 protein superfamily, controls seed germination, growth and biomass accumulation through ribosome-biogenesis protein interactions in *Arabidopsis thaliana*

**DOI:** 10.1186/1471-2229-14-37

**Published:** 2014-01-27

**Authors:** Emma W Gachomo, Jose C Jimenez-Lopez, Lyla Jno Baptiste, Simeon O Kotchoni

**Affiliations:** 1 Department of Biology, Rutgers University, 315 Penn St., Camden, NJ 08102, USA; 2 Center for Computational and Integrative Biology (CCIB), Rutgers University, 315 Penn St., Camden, NJ 08102, USA; 3 The UWA Institute of Agriculture, The University of Western Australia, 35 Stirling Highway, Crawley, Perth, WA 6009, Australia; 4 Department of Biochemistry, Cell and Molecular Biology of Plants, Estación Experimental del Zaidín, High Council for Scientific Research (CSIC), Profesor Albareda 1, Granada E-18008, Spain

**Keywords:** Arabidopsis, Gigantus1, Gene expression, Homology modeling, Docking

## Abstract

**Background:**

WD40 domains have been found in a plethora of eukaryotic proteins, acting as scaffolding molecules assisting proper activity of other proteins, and are involved in multi-cellular processes. They comprise several stretches of 44-60 amino acid residues often terminating with a WD di-peptide. They act as a site of protein-protein interactions or multi-interacting platforms, driving the assembly of protein complexes or as mediators of transient interplay among other proteins. In *Arabidopsis*, members of WD40 protein superfamily are known as key regulators of plant-specific events, biologically playing important roles in development and also during stress signaling.

**Results:**

Using reverse genetic and protein modeling approaches, we characterize GIGANTUS1 (GTS1), a new member of WD40 repeat protein in *Arabidopsis thaliana* and provide evidence of its role in controlling plant growth development. GTS1 is highly expressed during embryo development and negatively regulates seed germination, biomass yield and growth improvement in plants. Structural modeling analysis suggests that GTS1 folds into a β-propeller with seven pseudo symmetrically arranged blades around a central axis. Molecular docking analysis shows that GTS1 physically interacts with two ribosomal protein partners, a component of ribosome Nop16, and a ribosome-biogenesis factor L19e through β-propeller blade 4 to regulate cell growth development.

**Conclusions:**

Our results indicate that GTS1 might function in plant developmental processes by regulating ribosomal structural features, activities and biogenesis in plant cells. Our results suggest that GIGANTUS1 might be a promising target to engineer transgenic plants with higher biomass and improved growth development for plant-based bioenergy production.

## Background

In plants, growth development, cell patterning, yield, and biomass accumulation are controlled by functional genetic networks of several orders of magnitude that will require various complementary research approaches to uncover the basis of this functional genetic complexity. Using a combination of reverse genetic and computational protein modeling, we identified a protein, termed GIGANTUS1 (GTS1), a member of transducin/WD40 protein superfamily that regulates growth development in plants.

Transducin/WD40 repeat proteins are prominent features within proteins that mediate diverse protein-protein interactions, including those involved in scaffolding and the cooperative assembly and regulation of dynamic multi-subunit complexes [1,2]. The common and defined feature of these proteins are short ~40 amino acid motifs, typically ending by Trp-Asp sequence, and usually composed of a 7-8 bladed beta-propeller fold. However, a diversity of proteins has been found with 4 to 16 repeated units [3]. The low level of sequence conservation and functional diversity of WD40 domains make their identification difficult.

Repeated WD40 domains play central roles in biological processes such as cell division and cytokinesis, apoptosis, light signaling and vision, cell motility, flowering, floral development, meristem organization, protein trafficking, cytoskeleton dynamics, chemotaxis, nuclear export to RNA processing, chromatin modification, and transcriptional mechanism [4]. They act as a site for protein-protein interaction, where the specificity of the proteins is determined by the sequences outside the repeats themselves. Their functional importance resides largely on the protein surfaces. They serve as multi-interacting platforms in cellular networks for the assembly of protein complexes or mediators of transient interplay among other proteins [5]. Structural studies suggest that this property stems from their ability to interact with diverse proteins, peptides or nucleic acids using multiple surfaces, where the most common peptide interaction site of WD40 proteins is located on the top surface of the propeller close to the central channel [6].

Although WD40 proteins also are present in bacteria, e.g. *Thermomonospora curvata*[7] and *Cyanobacterium synechocystis*[8], WD40 domains are among the ten most abundant domain types across eukaryotic proteomes, and interactome studies suggest that they are among the most promiscuous interactors. Despite several WD40-containing proteins acting as key regulators of plant-specific developmental events, WD40 domains have been given less research attention compared to other common domains, for example kinase domains. Up to now, there have been no comprehensive 3D structural analyses of WD40 protein revealing the interacting partners and highlighting the relevance of WD40 domains mediating different biological functions [9]. Furthermore, in contrast to other members of the β-propeller family, and despite being crucial for and residing in enzymatic complexes, no WD40 protein has been reported to possess catalytic activity.

In order to fully understand the molecular- and structural-based regulatory mechanisms of GTS1 and the transducin/WD40 protein family, we carried out a comprehensive expression profiling, a mutational-based phenotypic characterization and a structural-based protein modeling of functional features of GIGANTUS1 in Arabidopsis. This is the first study in which the interacting partners of Arabidosis GTS1 protein have been identified. A comprehensive molecular and structural analysis based on protein homology modeling, and a docking interaction study to elucidate the functional mechanisms of GTS1 protein in regulating growth development in *Arabidopsis thaliana* were carried out. We described the phenotypic characterization of a *gts1* knockout mutant during growth development and assessed the GTS1-3D molecular structure and its docking features to uncover the regulatory relationship of GTS1 with other proteins.

## Methods

### Plant material and *GTS1* expression profiling analysis

*Arabidopsis thaliana* (ecotype Col-0) and *gts1* knockout mutant (T-DNA SALK_010647) from Arabidopsis Biological Research Center (ABRC) were used throughout this work. Appropriate seeds were sown on Murashige and Skoog (1 × MS) agar plates or soil and seedlings were allowed to grow under continuous illumination (120-150 μEm^-2^ s^-1^) at 24°C. Seedling samples were collected at different developmental stages for gene expression profiling. To analyze the expression of *GTS1* gene, total RNA was extracted with TRIzol reagent (Molecular Research Center) and then reversed transcribed using qScript cDNA Supermix (Quanta BioSciences, Gaithersburg, MD, USA) as previously described [10]. Thereafter, the cDNA was used as the template for PCR using gene-specific primers (Table 1), running 20 or 25 amplification cycles (linear range of amplification) unless otherwise noted [11]. The linear range of amplification was determined by running increasing cycle numbers and analyzing the amount of cDNA fragments. PCR fragments were separated on 1% agarose gels containing ethidium bromide. A cDNA fragment generated from ACTIN (AT3G18780) served as an internal control. T-DNA insertion in *GIGANTUS1* gene was PCR-confirmed using *GIGANTUS1* gene specific primers and T-DNA left border (LB) primer (Table 1). The expression of *GTS1* gene in *gts1* mutant background was analyzed by extracting total RNA from the *gts*1 homozygous line as above described and reverse transcribed into cDNA as previously described [10], and further used in expression analysis.

**Table 1:** Sequences of oligonucleotide primers used in this study

**Name**	**Primer sequence**	**Description**
GTS1-F1	5’GAGGAGCTGCAGGGTTATTT3’	For RT-PCR
GTS1-R1	5’CAAGACGGGTTAATCTGGGTAG3’	For RT-PCR
TDNA-LB	5’CCGTCTCACTGGTGAAAAGAA3’	For TDNA insertion
GTS1-F2	5’CTGAAACGGCAAATGGAAGAAG3’	For complementation test
GTS1-R2	5’CTATGTTGCTGGAAGTCGGAT3’	For complementation test
Act2-F	5’GCGGATCCATGGCTGAGGCTGATGATATTCAACC3’	For RT-PCR
Act2-R	5’CGTCTAGACCATGGAACATTTTCTGTGAACGATTCC3’	For RT-PCR

### *GTS1* database search and phylogenetic analysis

The *Arabidopsis thaliana GTS1* cDNA sequence (AT2G47790) was obtained from the Arabidopsis-TAIR website and used to perform a nucleotide BLAST (BLASTn) search of the *Oryza sativa* (rice) genomic sequence on the SALK Institute RiceGE2 web interface. The rice sequence identified as having the highest degree of homology to the Arabidopsis cDNA was downloaded and translated. The translated rice sequence was then aligned to the Arabidopsis protein sequence to validate the identification of the gene. The rice cDNA sequences were then used to perform a (BLASTn) search against the sequenced *Zea mays* genome on the MaizeSequence.org website. BLAST searches against the maize genome produced a list of BAC sequences that aligned to the query sequence. The BAC with the highest level of similarity was indicated on a genome map. The complete BAC sequence was downloaded and aligned to the *Oryza sativa* cDNA sequence using the NCBI BLAST (bl2seq) algorithm. Once the putative exons had been identified for a specific gene homolog, the exon start and end positions were manually corrected based on the canonical splice site donor/acceptor sequences and the overlapping sequence from one putative exon to the next. Each putative maize cDNA was then translated into a protein sequence. The GTS1 protein sequences from both *Oryza sativa* and *Zea mays* were aligned to the *Arabidopsis thaliana* sequence using the EMBOSS global pairwise alignment algorithm [12] to get the percent identity between the proteins. Next a BLASTp search of the *Homo sapiens* proteome in the NCBI database was performed using the *Arabidopsis thaliana* GTS1 protein sequence. From this search the Wdr89 sequence of *Homo sapiens* was identified as the most homologous protein. The Wdr89 sequence was used to retrieve (BLASTp) the *Rattus norvegicus* and the *Mus musculis* sequence homologs. Finally, a ClustalW2 alignment [13] was performed aligning all of the GTS1 protein sequences from *Arabidopsis thaliana, Oryza sativa,* and *Zea mays* with the Wdr89 protein sequences from *Homo sapiens*, Rattus *norvegicus*, and *Mus musculis*.

The conserved WD40 protein sequence from *Arabidopsis thaliana* was identified using ScanProsite and the Swiss-Prot/TrEMBLE databases. The identified WD40 sequence was used to perform a BLASTp search against plant proteomes on NCBI. From the list of potential WD40 containing proteins, the cDNA sequence of several *GTS1*-like proteins were downloaded from *Nicotiana benthamiana*, *Nicotiana tabacum, Pisum sativum*, *Phaseolus vulgaris*, *Medicago truncatula*, *Gossypium hirsutum*, *Lycopersicon esculentum*, *Solanum chacoense*, *Solanum lycopersicum*, and *Physcomitrella patens*. The collected plant WD40 containing cDNA sequences were combined with the *Arabidopsis thaliana*, *Oryza sativa*, and *Zea mays* cDNA sequences previously obtained and aligned using RevTrans version 1.4 [14]. Using MrAIC [15-20] model test software the best AIC model to use in constructing phylogenetic relationships was determined to be the General Transition Rate (GTR) with Gamma.

Phylogenetic trees were generated in MrBayes using four Markov Chain Monte Carlo runs with three cold and one hot chain each for 1,000,000 generations sampling every 100 generations. The burnin was determined to be within the first 4000 generations for each phylogeny. The first 10000 generations sampled were removed and a 50% consensus majority tree was constructed from the remaining trees. Trees were then drawn using TreeView version 1.6.6 and rooted using the *Physcomitrella patens* sequence as an out group.

### Sequences interaction database search

*Arabidopsis thaliana* GTS1, a Transducin/WD40 repeat protein (NCBI accession number AEC10888) was used as query to retrieve WD40 protein sequences from Uniprot (http://www.uniprot.org), and NCBI (http://www.ncbi.nlm.nih.gov) databases using BLAST tools (http://blast.ncbi.nlm.nih.gov/Blast.cgi).

GTS1 protein interaction network was obtained using the STRING v9.0 (http://string-db.org) database. STRING outcome gave the two most possible interacting protein counterparts for GTS1 protein, a 60S ribosome structural protein L19e (NCBI accession number AEE75864), and Nop16 (NCBI accession number AAP21378), a protein involved in 60S subunit ribosomal biogenesis.

### Functional domains analyses

GTS1 protein functional domains were studied by querying different structure-functional motifs and/or patterns databases such as Pfam v25.0 (http://pfam.sanger.ac.uk), Prosite (http://prosite.expasy.org/scanprosite), SMART v6.0 (http://smart.embl-heidelberg.de), Conserved Domain Database (CDD) v3.02, CDART (Conserved Domain Architecture Retrieval Tool) and CD-Search tools (http://www.ncbi.nlm.nih.gov/Structure/cdd/cdd.shtml), InterPRO v35.0 (http://www.ebi.ac.uk/interpro), ProDom release 2010.1 (http://prodom.prabi.fr/prodom/current/html/home.php), CATH v3.4 (http://www.cathdb.info), Superfamily v1.75 (http://supfam.cs.bris.ac.uk/SUPERFAMILY), PIRSF (http://pir.georgetown.edu), and functional searched by PANTHER (http://www.pantherdb.org). Similar analysis was performed for both interacting ribosomal protein counterparts, Nop16 and L19e.

### Secondary structure prediction

Secondary structural elements of the GTS1 protein were initially assessed for substructure conserved motifs by threading the sequences through the Protein Data Bank (PDB) (http://www.pdb.org) library using threading algorithm the Segmer [21]. These elements of the secondary structure were also confirmed by comparison with the results obtained with other additional 2-D structure servers: SSpro8 (Scratch Protein Predictor, http://scratch.proteomics.ics.uci.edu), NetSurfP ver. 1.1 (http://www.cbs.dtu.dk), and PSIPRED (http://bioinf.cs.ucl.ac.uk/psipred) fold servers. These secondary structure predictions were also performed for both interacting ribosomal protein counterparts, Nop16 and L19e.

### Structural templates searching

Protein sequences of GTS1, Nop16 and L19e were searched for homology in the Protein Data Bank (PDB). Homologous templates suitable for these three proteins were selected by BLASTp from the BLAST server (http://ncbi.nlm.nih.gov). The BioInfoBank Metaserver (http://meta.bioinfo.pl), which employs fold recognition for homology search, was also used for the selection of templates. The results obtained by previous methods were compared with the results obtained by Swiss-model server for template identification (http://swissmodel.expasy.org).

### Homology modeling

Homology modeling was performed by the I-TASSER server [22]. An initial structural model was generated and checked for recognition of errors in 3D structures using ProSA (http://prosa.services.came.sbg.ac.at/prosa.php), and for a first overall quality estimation of the model with QMEAN (http://swissmodel.expasy.org/qmean/cgi/index.cgi).

Energy minimization of the final structures was performed using GROMOS96 force field energy implemented in DeepView/Swiss-PDBViewer v3.7 (http://spdbv.vital-it.ch) in order to improve the van der Waals contacts and correct the stereochemistry of the model.

Each structure was assessed using the following softwares: QMEAN for quality, PROCHECK (http://www.ebi.ac.uk/thornton-srv/software/PROCHECK) for stereological corrections, ProSA, and ANOLEA (http://protein.bio.puc.cl/cardex/servers/anolea) for protein energy. The number of protein residues in the favored regions for each structure were calculated and visualized by The Ramachandran plot.

### Ligand-binding domains and conservational analysis

Ligand-binding sites in the 3D protein structures were analyzed using Cofactor software (http://zhanglab.ccmb.med.umich.edu/COFACTOR), to identify functional homology. Gene Ontology (GO) terms (The Gene Ontology project) were used to identify functional analogs based on the 3D built models, indicating the possible functions and biological pathway in which the proteins might be involved (http://www.geneontology.org).

Conservational analyses of the proteins were made by generating evolutionary related conservation scores using ConSurf server (http://consurf.tau.ac.il). Structural function conservation and key residues in the query proteins were identified by ConSeq server (http://conseq.tau.ac.il).

### Surface electrostatic potential analysis

The electrostatic Poisson-Boltzmann (PB) potentials for the surface amino acids of the structures were obtained using APBS software implemented in PyMol 0.99 (http://www.pymol.org) with AMBER99, and optimized with the Python software package PDB2PQR. Fine grid spaces of 0.35 Å were used to solve the linearized PB equation in sequential focusing multigrid calculations in a mesh of 130 points per dimension at 310.00 K. The dielectric constants were two for the proteins and 80.00 for water. The output mesh was processed in the scalar OpenDX format to render the isocontours and maps on the surfaces with PyMOL 0.99. Potential values are given in units of kT per unit charge (k Boltzmann’s constant; T temperature).

### Molecuolar docking analysis

The analysis of interactions between GTS1 with each of its ribosomal counterparts (Nop16 and L19e,) was performed using CLUSpro server [23]. During the workflow, backbone flexibility analysis was done using rigid-body ensemble docking with multiple structures derived from NMR, while the ZDOCK option for sampling at 6-degree rotational steps was used to obtain the decoys. The energies of docked conformations from protein-protein docking were evaluated by applying the Fast Fourier Transform (FFT) correlation approach.Docking scores were calculated by considering shape complementarity, desolvation, and electrostatics potential. The top docked conformations, along with their ZDOCK scores, were used as candidates of near-native structures, and for clustering of binding sites where the ligand was within 10 Å of its receptor.

After clustering, the ranked complexes were subjected to van der Waals minimization using CHARMM, and the protein-inhibitor structure with the best score was chosen as the most fitting model for GTS1-ribosomal interacting proteins.

## Results

### Molecular and structural analysis of GTS1

#### ***Expression profiling and phylogenetic analysis of GTS1***

**Figure 1:**
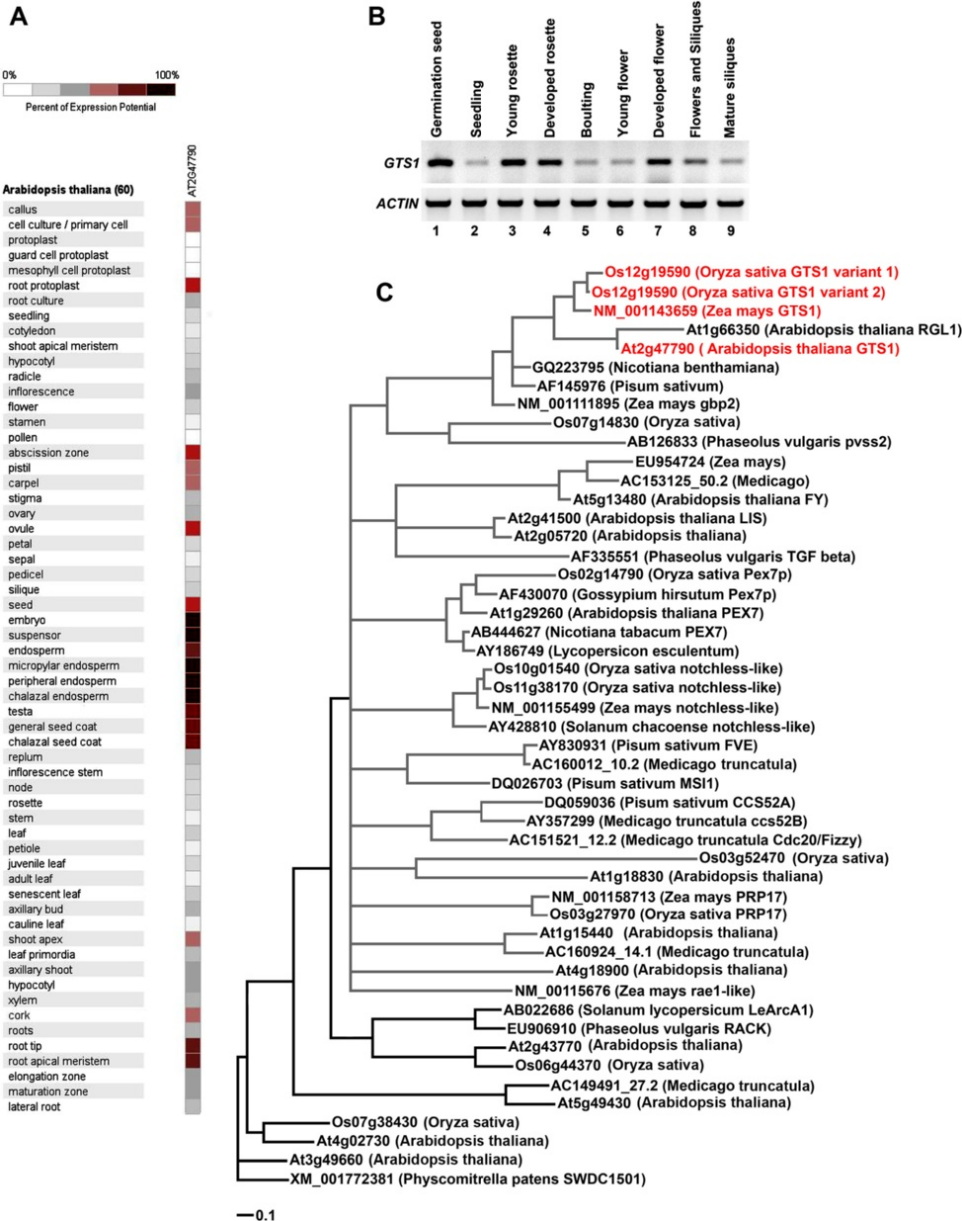
**Tissue specific expression profile and Phylogenetic analysis of GTS1. A)**. GENEVESTIGATOR-Microarray data showing highly expressed *GTS1* gene in embryo, root apical meristem, root tip, abscission zone and shoot apex [24]. **B)** Experimental expression analysis of GTS1 showing increased transcript accumulation in germinated seed, young and developed rosette leaves and developed flower. **C)**. Phylogenetic relationships between plant genes containing WD40 repeat domains. The GTS1 genes of *Arabidopsis thaliana*, *Oryza sativa*, and *Zea mays* (shown in red), belong to a subclade of the dominate clade (gray branches) containing most of the plant genes listed. Genebank accession numbers were used for all genes with the exception of *Arabidopsis thaliana* and *Oryza sativa* for which the Gene ID number from the SALK database were used.

The Arabidopsis *GTS1* is highly expressed during seed germination and particularly accumulating in embryo, ovule, and endosperm, (Figure 1A, B). It is abundantly expressed in meristemic regions, indicating its crucial role in regulating cell divisions (Figure 1A). The strong tissue (abscission zones) specific expression pattern of *GTS1* (Figure 1A) suggests its regulatory implication in plant growth developmental process (Figure 1A, B).

We confirmed that *GTS1* transcript accumulates in several major organs, including developing flowers, germinated seeds, young rosette leaves (Figure 1B). Microarray data analysis at different developmental stages also reveals an overlapping expression pattern of cell division/growth induced genes with *GTS1* (Additional file 1: Figure S1) as highlighted in Table 2[24]. These genes are involved in transcriptional and posttranscriptional processes, and various biochemical pathways (Table 2). On the basis of the high level of amino acid identity between Arabidopsis GTS1 and other well characterized WD40 protein homologs (Additional file 2: Figure S2), a phylogenetic analysis showed that Arabidopsis GTS1 is clustered with the rice and maize GTS1 (Figure 1C), thus indicating that they are more similar to one another than they are to other GTS1-WD40 repeat sequences (Figure 1C). This cluster belongs to a subclade of the dominant clade (Figure 1C, gray branches) containing most of the plant GTS1 protein homologs.

**Table 2:** **Tissue specific coexpressed clustering genes with *GTS1 *** **using GENEVESTIGATOR [**[24]**], and cross-verified in atted-II co-expression analysis (**http://atted.jp/**) revealing a strong specific correlative expression with ribosomal proteins (see in bold)**

**Gene locus**	**Function**
**AT2G47790 (GTS1)**	**Transducin/WD40 repeat-like superfamily protein**
At3g13460	Evolutionarily conserved C-terminal region 2
At5g04290	Kow domain-containing transcription factor 1
At1g43700	VIRE2-interacting protein 1
At1g01770	Protein of unknown function DUF1446 (InterPro:IPR010839)
At5g43720	Protein of unknown function (DUF2361)
At1g74040	2-isopropylmalate synthase 1
At2g32850	Protein kinase superfamily protein
At2g22090	RNA-binding (RRM/RBD/RNP motifs) family protein
At3g52120	SWAP (Suppressor-of-White-APricot)/surp domain-containing protein/D111/G-patch domain-containing protein
**At3g16780**	**Ribosomal protein L19e family protein**
At2g48120	Pale cress protein (PAC)
**At2g21580**	**Ribosomal protein S25 family protein**
At1g34180	NAC domain containing protein 16
At2g27880	Argonaute family protein
At1g62990	KNOTTED-like homeobox of Arabidopsis thaliana 7
At1g16430	Surfeit locus protein 5 subunit 22 of mediator complex

### GTS1 regulates seed germination and plant growth development

**Figure 2:**
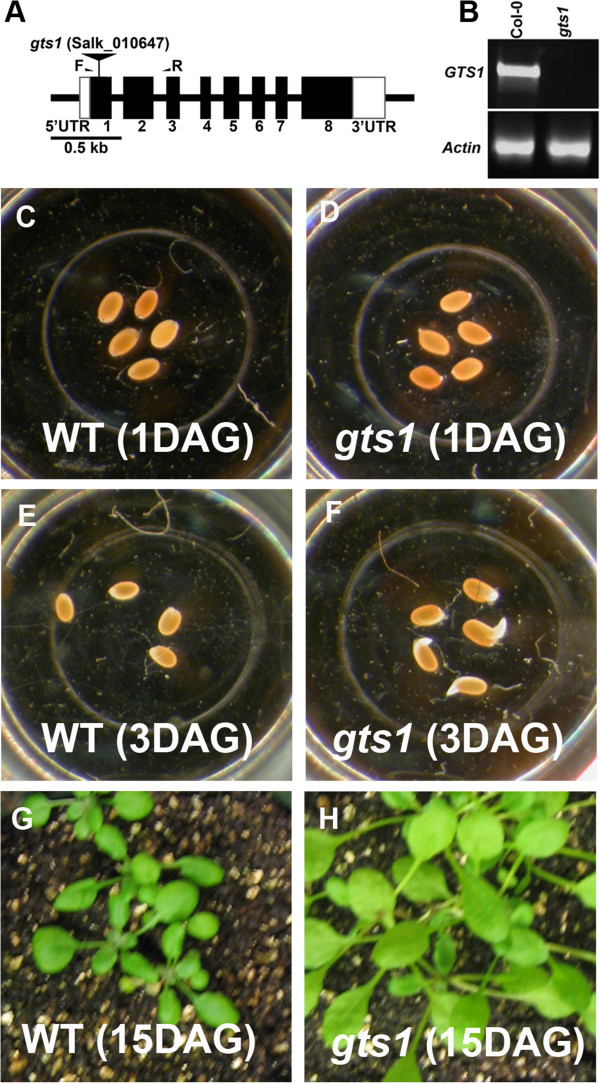
**Physical map of *GTS1 *knockout gene and phenotypic characterization of *gts1 *mutant. (A)** The *GTS1* gene with the positions of exons (numbered black rectangles) and introns (thick lines) are represented. The 5’ and 3’ untranslated regions are depicted in white rectangles. The location of the *gts1* T-DNA insertion is shown using an inverted black triangle. The names and locations of primers used for RT-PCR experiments are also indicated. Bar = 0. 5 kb. **(B)** The T-DNA insertion causes a knockout expression of the gene. The quality of the RNA and the loading control was assayed by monitoring ACTIN gene expression. **(C-F)** GTS1 negatively controls seed germination. *gts1* mutant germinated faster at 1 and 3 days after incubation in water **(D, F)** than the wild type **(C, E)**. **(G-H)** GTS1 controls biomass accumulation and growth development in Arabidopsis. **(H)**, Growth rate of *gts1* is faster than that of WT **(G)** at 15 DAG. *gts1* shows larger leaf area (biomass) **(H)** than WT **(G)**. DAG = Days after germination.

**Figure 3:**
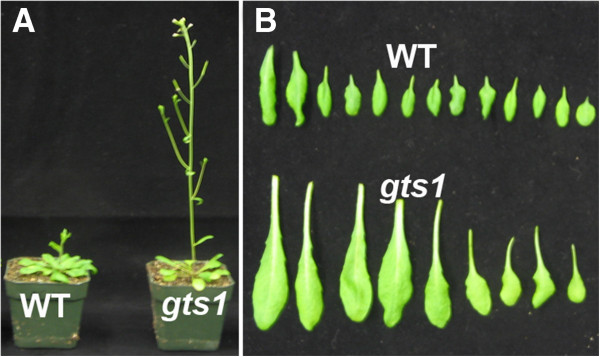
**Mutation in *GTS1 *gene promotes early flowering, growth development and biomass accumulation. A)** a faster growth of *gts1* mutant compared to WT is depicted with gts1 displaying a taller phenotype than WT. **B)***gts1* mutant flowers earlier than WT as depicted by a reduced number of *gts1* rosette leaves compared to WT at bolting time. Gts1 mutant accumulates higher cell biomass than WT as shown by a bigger overall *gts1* rosette leaf area compared to WT **(B)**.

In order to examine the role of GTS1 in plant growth development, we employed a reverse genetic approach using a SALK_TDNA knockout insertion (Salk_010647) of *GTS1* gene (Figure 2A) to investigate the effect of loss of GTS1 function in *gts1* mutant. The SALK_010647 (*gts1*) line harbored a T-DNA insertion in the first exon of *GTS1* gene (Figure 2A), which was PCR-confirmed by using the T-DNA-specific oligonucleotide primer LB1 and the *GTS1*-specific primers (Table 1). We next examined and confirmed the knockout *GTS1* mRNA transcript levels in *gts1* compared to WT using RT-PCR (Figure 2B). When compared to the wild type, the homozygous *gts1* mutant (n = 16) displayed a faster germination rate (Figure 2C-F), and a faster growth rate and higher biomass accumulation than the wild type (n = 16) (Figure 2G, H), indicating that GTS1 negatively regulates cell division, growth and overall biomass accumulation in meristemic regions. Furthermore, *gts1* mutant (n = 22) flowers earlier (5 days earlier) than the WT (n = 16) (Figure 3A), as demonstrated by a reduced number of rosette leaves (9 ± 0.6, n = 15) compared to the wild type (15 ± 0.5, n = 20) at bolting time (Figure 3B). The mutant (*gts1*) grows significantly taller than WT at the same day post germination (Figure 3A).

**Figure 4:**
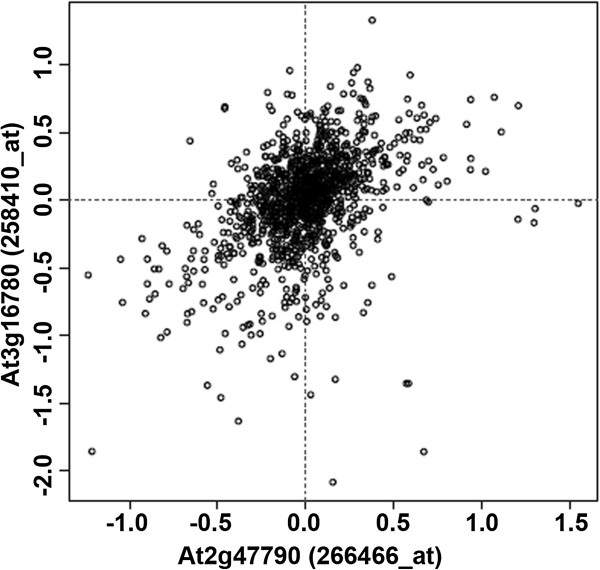
**Correlation of expression pattern between *GTS1 *and the ribosomal protein.** Samples whose contribution is more than 1.0 are outputted. The probe pair giving highest correlation is selected from all combination between the probes for the two loci, 266466_at (GTS1) and 258410_at (L19e) respectively. *Sample contribution score* is calculated as a product of z-scored expression values. The average of the score is the pearsons correlation coefficient.

In order to confirm that GTS1 is indeed responsible for these phenotypes, we performed a complementation test by RT-PCR amplifying a 1095 bp of *GTS1*-encoding sequence from WT cDNA (Table 1), cloned it into the *Sma*I site of the pROK2 vector [25] in front of CaMV 35S promoter-driven overexpression [26] and stably transformed *gts1* mutant background by the floral dip method [27]. As expected, the overexpression of *GTS1* in *gts1* mutant background was sufficient to abolishes the above described *gts1* phenotypes. The complemented line displayed a WT-like phenotype, indicating that this GTS1 is indeed responsible for the phenotypic characterization in *gts1* mutant. Unlike the other co-expression gene patterns (Table 2), we interestingly identified a strong gene-to-gene functional relationship between *GTS1* (AT2G47790) and the ribosomal protein *L19e* (At3g16780) (Figure 4). This data shows the detail of stability of co-expression between the 2 genes. The co-expression was supported by many factors, where the PCA correlation remained unchanged regardless of the growth parameter/factor considered. Both axes are relative gene expression values in base-2 logarithm against the averaged expression levels of each gene (Figure 4). These data argue for a strong gene-to-gene functional gene expression, suggesting that these two genes/proteins may physically interact to regulate/control biological processes in plants.

#### ***Searching for structural templates***

Since we confirmed that GTS1 regulates seed germination and growth development (biomass yield, and flowering time) in plants (Figures 2 and 3), we next examined the protein structure scaffold and interacting partners of GTS1 in accomplishing its functions. In order to study the physical interactions of GTS1 with other proteins in regulating growth development in plants, we performed a Protein Data Bank (PDB) search for GTS1 protein with known tertiary structure in PDB. The search yielded the crystal structures/PDB accession numbers 2h9l, 3iz6, 3ow8, 2gnq, and 1tbg, showing the highest sequence identity (22, 17, 16, 24, and 15%, respectively). The suitability of selected model was checked by BioInfoBank Metaserver, which returned 3D Jury scores (J-score) of 208.4 (2h9l), 200.2 (3iz6), 198.5 (3ow8), 205.7 (2gnq), and 201.9 (1tbg) for GTS1, respectively. In order to confirm the best possible templates to use for building the GTS1 structure, Swiss-Model server was used, finding high scores (64, 61, 63, 60, and 69) and very low E-values (1.5E-37, 2.1E-30, 4.9E-29, 2E-10, and 1.8E-43) for the templates 2h9l, (3iz6), 3ow8, 2gnq, and 1tbg respectively.

The same workflow was followed to obtain the best crystal templates in order 3D-structural protein model for the GTS1 ribosomal interacting partners. The search in the Protein Data Bank (PDB) for the protein L19e and Nop16 yielded the crystal structures of 3iz5, 3jyw, and 3u5e for L19e, and 2aje, 1w0t, 2juh, 2ckx, and 2roh for Nop16, showing comparable values in identity and suitability by BioInforMank Metaserver and Swiss-Model server analysis.

#### ***Quality of threading models***

To assess the quality of the protein models I*-TASSER and Procheck analysis* were performed. The I*-TASSER analysis* gave the accuracy parameters such as a C-score of -0.9, 0.60 ± 0.14 TM-score with 1848 decoys and 0.1467 of cluster density for GTS1, while P*rocheck analysis* revealed that the main chain conformations of GTS1 protein model were located in the acceptable regions of the Ramachandran plot. A majority of residues (77.6%) were in the most favorable regions, whereas 14.5% of the residues were placed in the allowed regions, and 6.5% were in generously allowed regions. Only 1.4% of the residues were present in the disallowed regions, respectively. The plot of ×1 versus ×2 torsion angles for each residue showed that most of the rotamers in GTS1 model was localized in low energy regions. iii) The *ProSa analysis* gave Z-scores of -5.71 for GTS1. The scores were within the range usually found for native proteins of similar size, i.e., -7.31, -4.02, -6.63, -7.93, and -7.33 for the templates 2h9l, 3iz6, 3ow8, 2gnq, and 1tbg crystal structures, respectively. iv) *QMEAN analysis* of GTS1 model revealed Q-values of 0.67. A quality factor of 0.793, 0.315, 0.71, 0.786, and 0.849 was estimated for the crystal structures of the templates 2h9l, 3iz6, 3ow8, 2gnq, and 1tbg, respectively, indicating that the GTS1 model is within the range of accuracy of the templates crystallographic structures. v) *Root mean square deviation* (RMSD) between GTS1 model and the crystal templates Cα backbones of the closed templates were 2.408 Å for 2gnq and 3.192 Å for 2h9l.

All of the above parameters were also determined for L19e and Nop16 protein models resulting in comparable structural quality values of our modeled L19e and Nop16 proteins.

#### ***3D structure of Arabidopsis GTS1***

**Figure 5:**
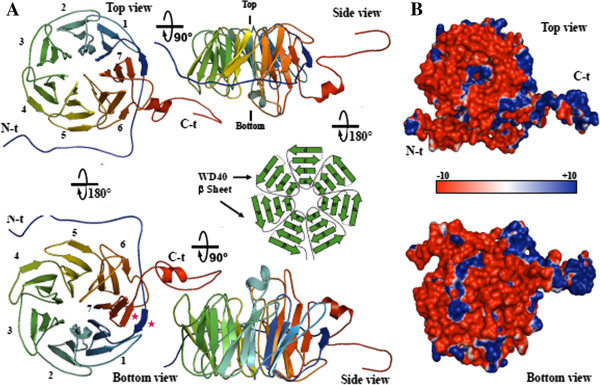
**Structure of GTS1, a WD40 repeat protein. A)** Top, bottom and side views of the seven-bladed β-propeller structure (most stable form) made by using PyMol software (http://www.pymol.org/), with the N-terminal and C-terminal regions in blue and red color, respectively. A depicted model is included to show the basic WD40 β-sheet structures conformation of the cylinder structure with a tunnel-like structure in the centre that communicate both top and bottom sides. **B)** 180° rotated views of the electrostatic potential representation on the GTS1 protein surface. The surface colors are clamped at red (-10) or blue (+10).

We obtained the best structural models of this newly described Arabidopsis WD40 repeat protein, GTS1, based on homology modeling (Figure 5). The 3D structure of Arabidopsis GTS1 belongs to the transducin/WD40 repeat protein family because it shares all the structural characteristics of WD40 proteins (Figure 5A), which agree with the general crystal structure of 2h9l or 2gnq template. In general, the structure can be visualized as a short, open cylinder where the strands form the walls. At least four repeats are required to form a β-propeller. In our case, GTS1 contains 7 WDs, where the final and last (i.e., the N- and C-terminal) WDs participate in the same β-propeller (Figure 5A), potentially reinforcing the structure. Despite the low amino acid sequence identity across species, a relatively good conservation of the overall fold (Cα carbon chain) of this protein is found among plant species and eukaryotes in general [4,28].

Surface electrostatic potential analysis (Figure 5B) reveals several prominent positively charged residues (blue regions), predominantly in the walls of the cylinder (tunnel) and C-terminal arm. The environment of this protein is essentially negatively charged (red regions) (Figure 5B), as highlighted by the Poisson - Boltzmann electrostatic potential. By assigning a value of +1 to basic residues (R, K) and -1 to the acidic residues (D, E), net charge of protein was calculated to be -22 for GTS1. The central tunnel of the GTS1 structure exhibited a predominantly positive charge in the top view. Comparison between GTS1 and other WD40 repeat proteins, such as templates 2gnq and 2h9l did not exhibited large differences in the general topology as it was further confirmed by the RMSD value of 2.408 Å and 3.192 Å, respectively, whereas significant differences were found in particular regions of the proteins such as the N-terminal, and C-terminal regions.

### Conservational and functional/ligand-binding site analysis

**Figure 6:**
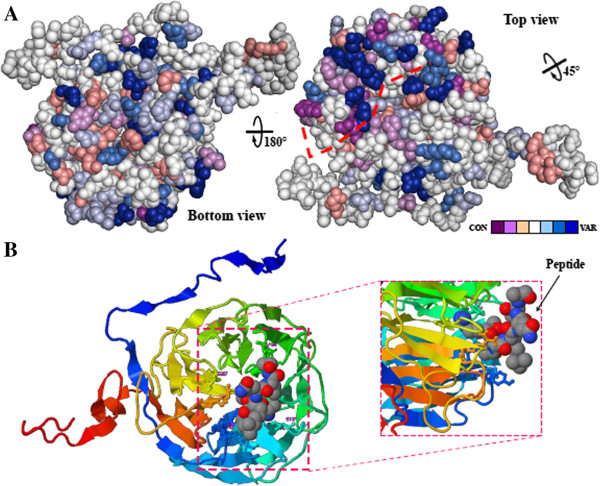
**Conservational and ligand binding domain analysis of GTS1, a WD40 repeat protein. A)** Consurf-conservational analysis of GTS1 protein showed in two individual views rotated 180°. The conserved and variable residues are presented as space-filled models and colored according to the conservation scores. The interacting area of the protein with ribosomal counterparts has been highlighted by a red discontinue line. **B)** Detailed view of the ligand-binding area of GTS1 with a peptide and the spatial distribution of the interacting residues in a detailed view.

The conservational and ligand-binding or functional features of GTS1 were analyzed (Figure 6). Surfaces of Arabidopsis GTS1 (rotated 180°) showing the conservation index of residues are depicted in Figure 6A. Consurf conservation analysis showed that GTS1 cylinder-like β-propeller structure is quite well conserved, especially the residues located in the core of the structure (Figure 6A). The N- and C-terminals of the protein are other most conserved regions of the protein, besides the core area which has a major role in maintenance of the protein structure [9,29]. This distribution of the core conserved region and surface variable residues helps maintain a similar overall fold among WD40 repeat proteins, but also produces differences observed in terms of a multiple protein interactions along WD40 repeat protein, where it is indicated by a discontinued red line for both ribosomal proteins in the GTS1 structure (Figure 6A).

Figure 2B shows a general view of the amino acids in the tunnel-like structure holding up the interacting peptides. GTS1 exhibit the putative active (most representative ligand-binding) site located in the center of the structure (Figure 6B, top view), which normally is another interacting polypeptide, containing several conserved but also few variable amino acids. A detailed view, showing the spatial distribution of residues responsible for the conformation of the ligand binding domain surrounding the ligand and directly implicated in this interaction are **Y43**, *V44*, **F45**, S61, N87, **S107**, **F134**, **V194**, S267, *R329*, and the peptide chain substrate bound to the tunnel-like structure (Figure 6B, detailed view at the right side). Conservational analysis of WD40 repeat proteins with significant close identity to Arabidopsis GTS1 among other species returned a large number of highly variable (bold) and small number of conserved (*italic*) residues as written above. Conformational predictions indicate that the peptide (ligand) is projected to and partially located inside the tunnel-like structure of GTS1.

**Figure 7:**
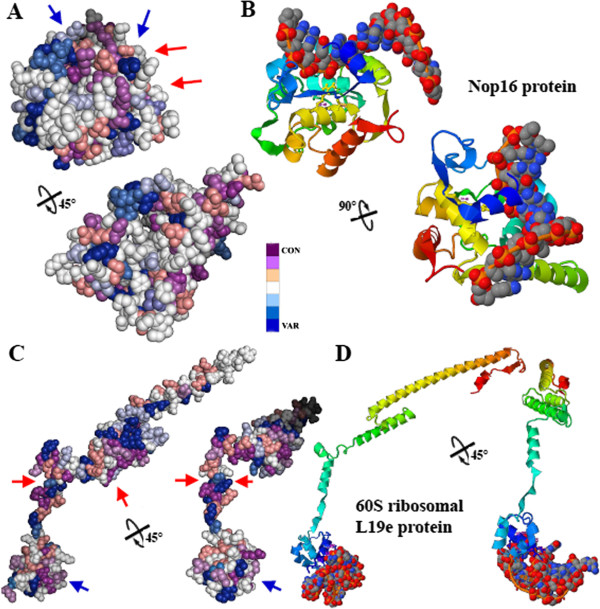
**Conservational and ligand binding domain analysis of ribosomal Nop16 and L19e proteins. A)** Consurf-conservational analysis of Nop16 protein showed in two individual views rotated 45°. The conserved and variable residues are presented as space-filled models and colored according to the conservation scores. The interacting areas of the protein with GTS1 protein and ribonucleic acid have been highlighted in red and blue color, respectively. **B)** Detailed views of the ligand-binding area of Nop16 protein with a chain of ribonucleic acid and the spatial distribution of the interacting residues depicted in both detailed views. **C)** Consurf-conservational analysis of L19e protein showed in two individual views rotated 45°. The conserved and variable residues are presented as space-filled models and colored according to the conservation scores. The interacting areas of the protein with GTS1 protein and ribonucleic acid have been highlighted in red and blue color, respectively. **D)** Detailed views of the ligand-binding area of L19e protein with ribonucleic acid and the spatial distribution of the interacting residues depicted in both detailed views.

The conservational and functional analysis of the ribosomal L19e and Nop16 proteins are depicted in the Figure 7. Consurf conservation analysis in the Figure 6A showed that Nop16 protein is well conserved, especially in the interacting surface region (red arrows, and Figure 7B) and the nucleic acids (blue arrow). Only few light and deep blue residues are around the surface of the protein. Ligand-binding analysis of this protein showed in Figure 7B highlights the area of the Nop16 protein where the interaction with nucleic acids takes place. This area is located in a cleft integrated by the N-terminal α-helix. The residues implicated in the interaction between the protein and the nucleic acid are *L40*, *M41*, *T142*, and *R146*, resulting in four not well conserved amino acids (*italic*).

Consurf conservation analysis showed in the Figure 7C highlights an equally distributed number of conserved and variable residues along the surface of L19e protein. The area of interaction with GTS1 seems not to be as well conserved as its counterpart Nop16. Few variable residues (blue color) are located in the area of interaction with the N-terminal tail of GTS1 (red arrows in Figure 7C). In addition, among the surface directly implicated in the interaction with the ribonucleic acid (blue arrows in Figures. 7C, D), S3, K5, *I6*, **R9**, L10, N36 seems to be well conserved, since only one residue, I6, was found to be variable (italic), and the rest exhibit an average index of conservation or highly conserved like R9 (bold).

### GTS1 interaction mechanism: molecular docking analysis with ribosomal counterparts

In order to get insights into the GTS1 regulatory/multi-interacting mode with other proteins, we analyzed the conformational interaction between GTS1 and two interacting partners involved in the structure and biogenesis of ribosomes in Arabidopsis. This analysis was carried out by molecular docking, using newly modeled structures of the two ribosomal proteins.

**Figure 8:**
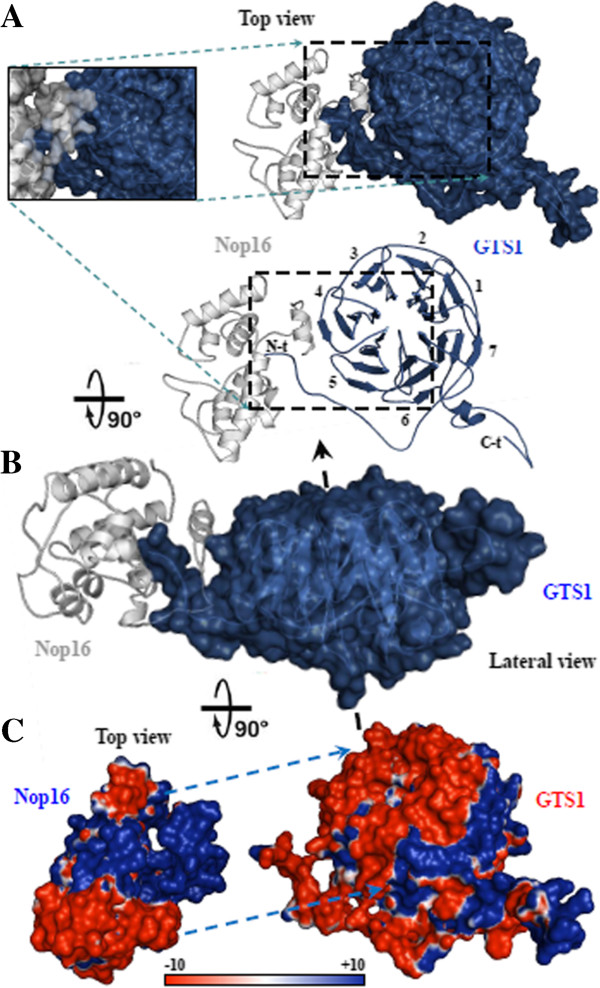
**Analysis of the interaction between GTS1, a WD40 repeat and Nop16 proteins. A)** The complex between GTS1 (blue surface and cartoon representation) and Nop16 (white/gray surface and cartoon representation) from the top view. **B)** Surface/cartoon structure rotated 90° in blue (GTS1) and white/gray (Nop16) are depicted, and highlight the large interacting surface between both proteins. **C)** Electrostatic potential depicted in both interacting partners, where has been highlighted both areas involved in the interaction by light-blue discontinue arrows. The surface colors are clamped at red (-10) or blue (+10).

Figure 8 shows the mode of interaction between Arabidopsis GTS1 and the nucleolar protein 16 (Nop16), involved in the biogenesis of 60S ribosomal subunit. The binding mechanism occurs through the formation of a stoichiometry complex 1:1 between both proteins. A detailed view of this interaction is depicted in the magnified views displayed in Figure 8A, where C-terminal arm of GTS1 is located inside the cavity made by N-terminal α-helix of Nop16 and neighboring helices, in addition to the direct interaction of this N-t α-helix with the β-propeller 4 structure of GTS1. The N-t α-helix of GTS1 is covered completely by the Nop16 cleft, therefore preventing access by other interacting partners to this area. On the other hand, it also partially impedes the interactions that are necessary for binding the nucleic acid to Nop16 by stereological impediment. The interaction is non-covalent, thus it may be reversible by increasing salt concentration and/or pH to alkaline conditions. Figure 8B shows the large area of interaction covered by the Nop16 molecule in the GTS1 in a perpendicular (90º) view.

With regard to energy, the electrostatic potential analysis for the contact surface of both structures exhibited a highly compatible fingerprint distribution of opposite charges, i.e., positive (in the contact area of Nop16 protein), and negative (mainly for GTS1) (Figure 8C). There are not large areas with hydrophilic character in the contact surface between both proteins, and the formation of the complex may be mediated by a high number of direct and water-mediated H-bonds.

**Figure 9:**
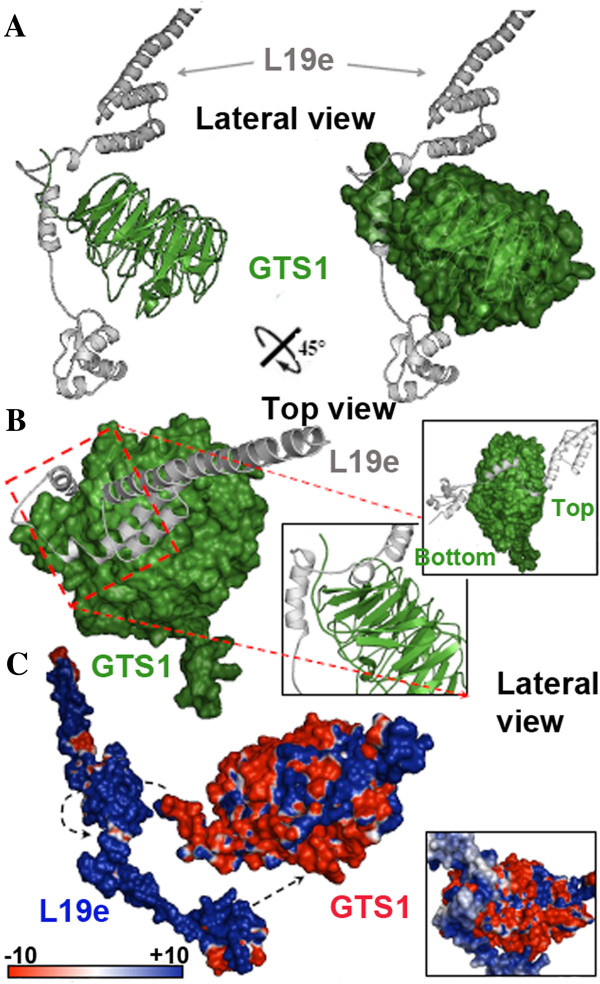
**Analysis of the interaction between GTS1, a WD40 repeat and L19e proteins. A)** The complex between GTS1 (green surface and cartoon representation) and L19e (white/gray cartoon representation) from a lateral view. **B)** Surface/cartoon structure rotated 45° in green (GTS1) and white/gray (L19e) are depicted, and highlight the two interacting surfaces between both proteins. **C)** Electrostatic potential represented in both interacting partners, where has been highlighted both areas involved in the interaction by black discontinue arrows. A detailed view of this interaction has been depicted. The surface colors are clamped at red (-10) or blue (+10).

Figure 9 shows the mode of interaction between Arabidopsis GTS1 and the 60S ribosomal protein L19e. The binding mechanism also occurs through the formation of a stoichiometry complex 1:1 between both proteins. A detailed view of this interaction is depicted in the magnified view (Figure 9A), where the N-terminal area of GTS1 hugs the thin structure of L19e, and the neighboring α-helix contacts the β-propeller 4 of GTS1. Additionally, the N-terminal area of the L19e protein directly interacts with the residues of the central bottom side of the GTS1 tunnel, leaving the central top side of the tunnel free as detailed in Figure 8B. The same area of L19e protein is also involved in the interaction with ribonucleic acid (Figure 7D). The electrostatic potential analysis for the contact surfaces of both structures exhibited a highly compatible fingerprint of opposite charges (positive in the contact area of L19e protein and negative in the contact surface of GTS1) (Figure 9C).

## Discussion

GIGANTUS1 is here described to be very important in regulating plant growth development (seed germination, faster growth, flowering time, and biomass accumulation) (Figures 1, 2 and 3). This is the first time that a mutation in *GTS1* has been implicated in early germination, growth and development in *Arabidopsis thaliana*. The molecular mechanism by which GIGANTUS1 regulates plant growth development is still unknown. As a member of WD40 protein family, GTS1 is expected to play central roles in different biological processes including cell division and cytokinesis, flowering, floral development, cytoskeleton dynamics, nuclear export to RNA processing, transcriptional mechanism, and protein-protein interactions [4]. We postulate that GIGANTUS1 might primarily function as a site for protein-protein interaction or mediator of transient interplay among other proteins to regulate different biological processes in plants. The development of protein complexes involves regulatory interactions that are mainly controlled by scaffolding proteins, such as WD40 repeat motifs. These motifs are important features of diverse protein-protein interactions [4], providing an unbending platform for interactions of proteins with other cellular components and controlling therefore several vital functions of the cell, such as signaling cascades, cellular transport and apoptosis [29-31].

The WD40 domains in GTS1 protein are shown to contain seven or multiples of seven repeats forming a highly stable β-propeller structure (Figure 5A). The 7-fold β-propeller is the most stable β-sheet geometry characterizing the resolved WD40 structures and also used to identify WD40 proteins [32]. However members of this protein family have been also found to contain as high as sixteen repeats [33]. Proteins with less than 7 repeats form an incomplete β-propeller structure and require additional WD-repeats from their neighbors to stabilize themselves, making dimers [34]. There is no apparent folding order for each repeat and the order in which repeats fold might vary among different WD40 proteins, or even within the same protein [35]. These proteins are known be involved in light signaling/photomorphogenesis and flowering [36], auxin response and cell division [37], in meristem maintenance [38], floral development [39], seed development and flowering [40], chromatin-based gene silencing and organogenesis [39], protein turn-over, microtubule dynamics, phospholipid binding and vesicle coating [4]. This justifies the great deal of research interest in the WD40 protein superfamily across plant species. Our data (Figures 2 and 3) suggest that GTS1 belongs to the WD40 protein subfamily regulating auxin response and cell division [37], meristem maintenance [38], floral development [39], seed development and flowering [40]. In this study the Arabidopsis GTS1 ligand-binding domain (main functional domain) lies mainly on the top surface residues, which integrates parts of β-propeller domain (Figure 6B). However, our data revealed that GTS1 WD40 propellers have three distinct surfaces available for interactions: the top region of the propeller, the bottom region (Figure 9), and the circumference [4,5] (Figures 8 and 9), suggesting the multi-functional properties of GTS1 protein through protein-protein interactions. Indeed, protein-protein and protein-peptide interactions involved the entry site of the central channel of the β-propeller (Figure 6B), where the majority of interaction partners (including small molecules) bind [5]. N- or C-terminal extensions of GTS1 run parallel to the tunnel-like structure, which form the complete 7 WD40 repeat domains, making them accessible for interaction with other partners (Figures 8 and 9). WD40 domains can thus act as large interaction platforms for multiple protein interactions. In comparison to other domains, the proteins containing WD40 motif are components of several interaction pairs [41,42], and act as scaffolds for larger complex assemblies. This work represents the first time the 3D-structural and molecular features of the GTS1 protein in plants have been examined.

To better understand what area of the GTS1 plays an important role in complex formation for functional conservation across plant species, we identified two fundamentally conserved regions; the first, located on the top rim, constituted by the residues composing blades 4 to 7, including N- and C-terminal arms; and the second large conserved surface is located on the bottom of the propeller and is mainly composed of blades 1, 2 and 3 (Figure 6A). These regions represent potential protein-protein interaction sites [43].

In general, plant and particularly Arabidopsis-WD repeat proteins are strongly conserved. Most of these proteins are components of basic cellular machinery regulating plant-specific processes. An interesting question arises as to how these proteins evolved into their specific cellular roles. One of the key functional processes of WD proteins is the biogenesis of eukaryotic ribosomes, a highly regulated and dynamic process that begins in the nucleolus with transcription of rRNA precursor (pre-rRNA) and rapidly packaged into the 90S ribonucleoprotein particle containing ribosomal proteins, non-ribosomal proteins, and snoRNA-containing ribonucleoprotein particles (snoRNPs). The 90S pre-RNPs are converted into 43S and 66S ribosome assembly intermediates, which ultimately give rise to mature 40S and 60S ribosomal subunits [44]. It is well known that ribosome biogenesis is driven by a large number of pre-ribosomal factors that associate with and/or dissociate from the pre-ribosomal particles along the maturation pathway. Although there has been much progress to identify ribosome assembly intermediates and their protein and RNA constituents [45], the information about the architecture of these pre-rRNPs is scarce. It is unclear which proteins are the nearest neighbors within the assembled ribosomes and to what extent neighboring molecules function together.

WD40 protein-protein interaction motifs represent excellent candidates to mediate interactions in the multiprotein subcomplex comprising a neighborhood in assembling ribosomes because of their protein-protein multi-interacting versatility. More than 70 trans-acting factors required for ribosome assembly have been identified [46], as well as 80 additional assembly factors present in pre-ribosomes [47]. Therefore, such WD40-containing proteins may nucleate the assembly of pre-ribosomes by interacting sequentially or simultaneously with other assembly factors or ribosomal proteins. Among the assembly factors, 17 proteins were found to contain WD40 motifs [48]. Many of the annotated ribosome biogenesis-WD40 repeat proteins were shown to directly interact with, or regulate the levels of other proteins [49] or to be components of multiprotein subcomplexes. Yeast WD40 protein Ytm1 is a constituent of 66S pre-rRNPs, whose depletion resulted in a deficiency of 60S ribosomal subunits [50]. Its homologue, mammalian WDR12 functions in the maturation of the 60S ribosomal subunit. WDR12 forms a stable complex with a novel member the nucleolar proteins Pes1 and Bop1 (Pe- BoW complex), which are crucial for processing of the 32S precursor ribosomal RNA (rRNA) and cell proliferation [36]. Interestingly, a potential homologous complex of Pes1–Bop1–WDR12 in yeast (Nop7p-Erb1p-Ytm1p) is involved in the control of ribosome biogenesis and S phase entry [51].

The yeast WD40 repeat protein Mak11 that modulates a p21-activated protein kinase function is an essential factor in nuclear maturation of 60S ribosomal subunits and its depletion led to a cell cycle delay in G1, indicating an early step nucleolar role of Mak11 in ribosome assembly. Another sub-complex, transiently associated with late, nuclear pre-60S precursors, is composed of four proteins and contains Ipi3 as a WD40 repeat member [52].

In this study, a new interacting counterpart, the Arabidopsis Nop16 protein was identified as a potential ribosome biogenesis factor in plants, which could be implicated in formation of the 60S ribosomal precursor. This process may be regulated by an interaction with GTS1 (Figure 8). This interaction was studied using a docking analysis that showed a stable interaction between GTS1 and Nop16, involving the N-terminal tail and the 4^th^ blade of the first partner, and a cleft formed in the second ribosomal factor by the N-terminal α-helix and the neighboring secondary elements. Another ribosomal protein (L19e protein) was found to interact with GTS1. L19e protein is implicated in the structural stability of ribosome. The interacting area between GTS1 and L19e is very close to that of Nop16 and GTS1 interacting area (Figure 8). This suggests that the interacting mechanism of regulating the biogenesis nucleolar factor Nop16 and the structural ribosome factor L19e may be competitive. Therefore both steps in the 60S ribosomal subunit formation, structural maturation and stabilization may be separate in the time and/or different cellular compartments.

In support of our data are two other examples of WD40 repeat ribosome biogenesis factors, Rrb1 and Sqt1, which interact directly with ribosomal proteins for 60S ribosomal subunit assembly. Rrb1 interacts with the ribosomal protein Rpl3 in the nucleus and regulates its levels [53], and Sqt1 interacts with Rpl10 in the cytoplasm [54]. Both proteins have a role in the association of the corresponding ribosomal protein with the nascent 60S ribosomal subunits and might regulate the levels of the corresponding ribosomal protein. Other WD40 repeat proteins have been implicated in the formation and stabilization of the small ribosomal subunit 40S. Yeast RACK1 regulates the translation initiation by recruiting PKC to the ribosome [55,56]. Four RACK1 orthologs identified in *Arabidopsis thaliana* may have a similar activity [57]. These interactions could provide a mechanism to regulate translation activities of ribosome populations programmed with specific mRNAs [58].

## Conclusions

The present study provides substantial evidence for the role of GIGANTUS1 in controlling seed germination, faster growth and biomass accumulation in plants. The gene is mainly expressed in meristemic regions and is therefore important in cell division. It is postulated to regulate growth development through diverse protein-protein interactions, including those involved in scaffolding and dynamic multi-subunit complexes such as the ribosomal protein biogenesis, stability and activity. Given its rich interaction surfaces, GTS1 functions probably as an adaptor in many different protein complexes or protein-DNA complexes in very diverse cellular processes that needs further research investigation. Our modeling data suggests that GTS1 mediates molecular recognition events mainly through the smaller top surface of domain, which comprises three residues forming a transient complex with other peptides. It would be interesting to further investigate GIGANTUS1 knockout genes in agronomically important crops with the aim of improving crop yield and biomass accumulation for sustainable plant-based biofuel production.

## Competing interests

The authors declare that they have no competing interests.

## Authors’ contributions

SOK conceived the study. SOK, JCJ-L, EWG wrote the paper. SOK, JCJL, EWG, LJ performed the study. SOK JCJ-L, EWG, analyzed, discussed and assessed the data. JCJ-L, EWG, SOK contributed reagents/materials/analysis tools. All the authors approved the final manuscript.

## Supplementary Material

Additional file 1: Figure S1 Hierarchical clustering microarray expression analysis of GTS1 and other selected tissue specific genes. Depicted squares display genes with similar tissue specific expression pattern with GTS1 (see Table 2 for detail description). Data analysis was retrieved from Genevestigator [24]. Click here for file

Additional file 2: Figure S2 The plant GTS1 proteins are most similar to the animal Wdr89 protein. ClustalW alignment of the plant GTS1 proteins from *Arabidopsis thaliana* (AtGTS1), *Oryza sativa* (OsGTS1), and *Zea mays* (ZmGTS1), with the animal Wdr89 proteins from *Homo sapiens* (HsWdr89), *Ratus norvegicus* (RnWdr89), and *Mus musculus* (MmWdr89) show several conserved residues across the entire length of the protein (gray shaded residues). *Oryza sativa* (OsGTS1) and *Zea mays* (ZmGTS1) share 53.8% and 52.7% identity with AtGTS1 protein, respectively. WD40 repeat domains are underlined for all three plant GTS1 proteins. Click here for file
