# Supplementary figures and images for: GIGANTUS1 (GTS1), a member of Transducin/WD40 protein superfamily, controls seed germination, growth and biomass accumulation through ribosome-biogenesis protein interactions in Arabidopsis thaliana
Source: BMC Plant Biol. 2014 Jan 27;14:37. doi: 10.1186/1471-2229-14-37 (PMC3914372; doi:10.1186/1471-2229-14-37)

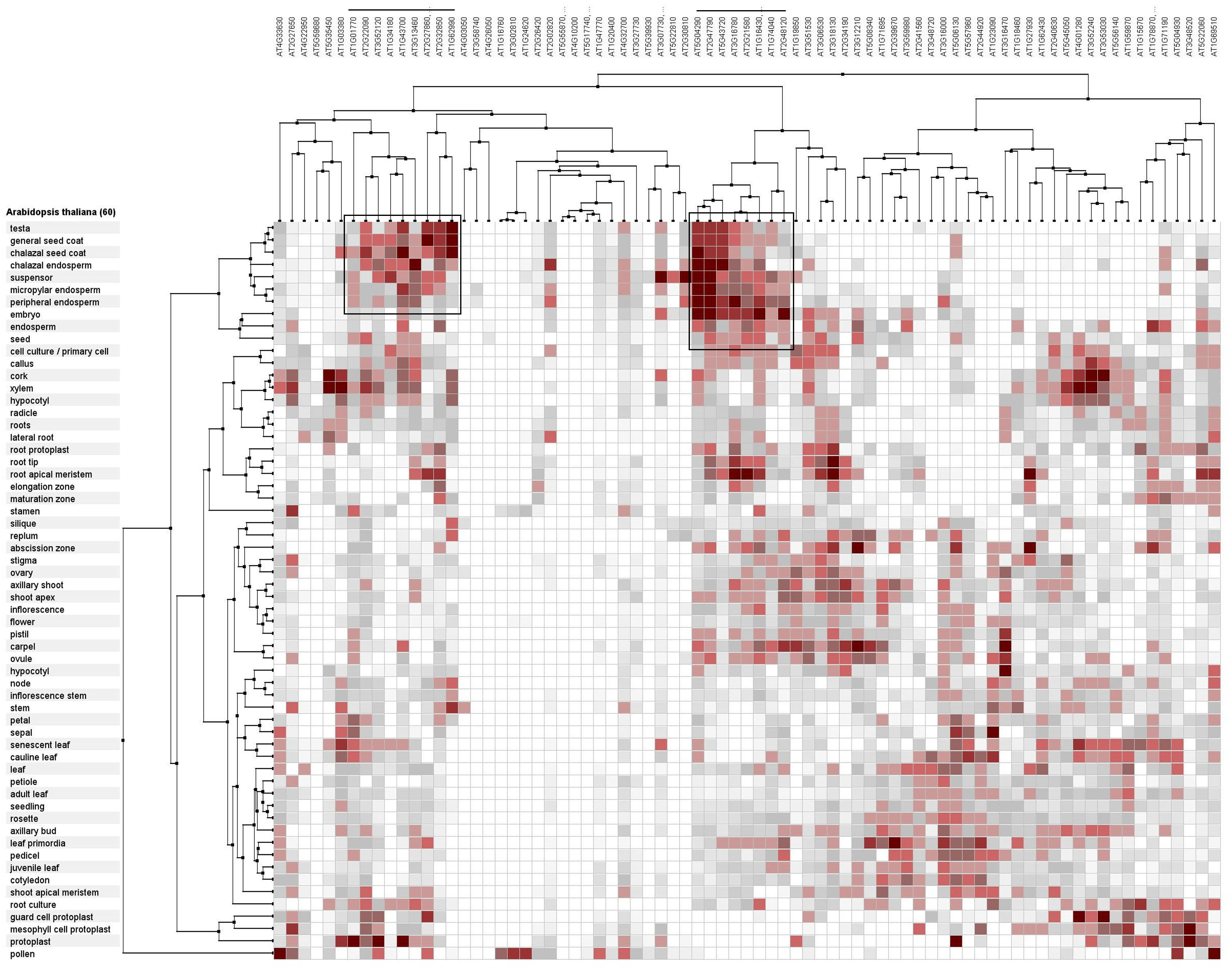

Supplement: Additional file 1: Figure S1 — Hierarchical clustering microarray expression analysis of GTS1 and other selected tissue specific genes. Depicted squares display genes with similar tissue specific expression pattern with GTS1 (see Table 2 for detail description). Data analysis was retrieved from Genevestigator [24]. [file 1471-2229-14-37-S1.jpeg]

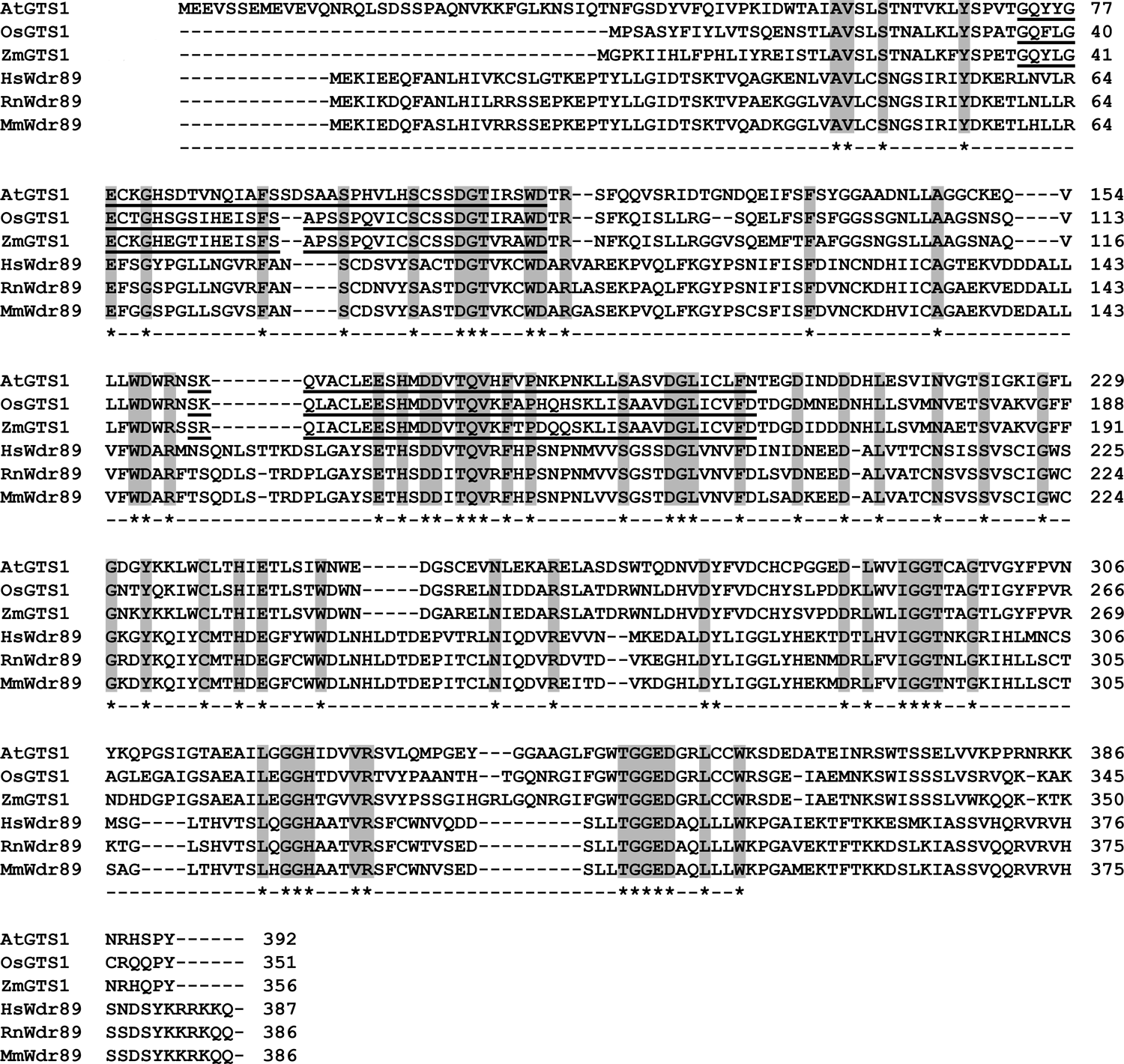

Supplement: Additional file 2: Figure S2 — The plant GTS1 proteins are most similar to the animal Wdr89 protein. ClustalW alignment of the plant GTS1 proteins from Arabidopsis thaliana (AtGTS1), Oryza sativa (OsGTS1), and Zea mays (ZmGTS1), with the animal Wdr89 proteins from Homo sapiens (HsWdr89), Ratus norvegicus (RnWdr89), and Mus musculus (MmWdr89) show several conserved residues across the entire length of the protein (gray shaded residues). Oryza sativa (OsGTS1) and Zea mays (ZmGTS1) share 53.8% and 52.7% identity with AtGTS1 protein, respectively. WD40 repeat domains are underlined for all three plant GTS1 proteins. [file 1471-2229-14-37-S2.tiff]
